# Supplementary material for: MetaRibo-Seq measures translation in microbiomes
Source: Nat Commun. 2020 Jun 29;11:3268. doi: 10.1038/s41467-020-17081-z (PMC7324362; doi:10.1038/s41467-020-17081-z)
Supplement: Supplementary file 10 — Supplementary Data 7 [file 41467_2020_17081_MOESM10_ESM.zip › File2/Confidence_High_Taxonomy/278094_out.krona.html]

Javascript must be enabled to view this page.

members
magnitude
magnitudeUnassigned
count
unassigned
taxon
rank

278094\_out

3

superkingdom
2
3

3
1239
phylum

3
186801
class

186802
3
order

541000
3
family

3
1263
genus

1
40519
species

SRS146812\_contig\_number\_20623


SRS014979\_contig\_number\_2488SRS144183\_contig\_number\_19109
species
1638786
2
